# Treatment of Nonalcoholic Steatohepatitis in Adults: Present and Future

**DOI:** 10.1155/2015/732870

**Published:** 2015-03-18

**Authors:** S. Gitto, G. Vitale, E. Villa, P. Andreone

**Affiliations:** ^1^Dipartimento di Gastroenterologia, Azienda Ospedaliero-Universitaria and Università di Modena e Reggio Emilia, 41125 Modena, Italy; ^2^Dipartimento di Scienze Mediche e Chirurgiche, Università di Bologna e Dipartimento dell'Apparato Digerente, Azienda Ospedaliero-Universitaria di Bologna, Policlinico Sant'Orsola Malpighi, 40138 Bologna, Italy

## Abstract

Nonalcoholic steatohepatitis has become one of the most common liver-related health problems. This condition has been linked to an unhealthy diet and weight gain, but it can also be observed in nonobese people. The standard of care is represented by the lifestyle intervention. However, because this approach has several limitations, such as a lack of compliance, the use of many drugs has been proposed. The first-line pharmacological choices are vitamin E and pioglitazone, both showing a positive effect on transaminases, fat accumulation, and inflammation. Nevertheless, vitamin E has no proven effect on fibrosis and on long-term morbidity and mortality and pioglitazone has a negative impact on weight. Other drugs have been studied such as metformin, ursodeoxycholic acid, statins, pentoxiphylline, and orlistat with only partially positive results. Among the emerging treatments, telmisartan is particularly interesting as it seems to have an impact on insulin resistance, liver steatosis, inflammation, and fibrosis. However, the pathogenesis of steatohepatitis is highly complex and is determined by different parallel hits; indeed, the association of different drugs that act on various levels has been suggested. In conclusion, lifestyle intervention should be optimised and the associations of different drugs should be tested in large studies with long-term outcomes.

## 1. Introduction

### 1.1. Definition and Aim

We define “nonalcoholic fatty liver disease (NAFLD)” as hepatic steatosis that accounts for more than 5–10% of the total hepatic weight. To make an accurate diagnosis, imaging or histological techniques are necessary, and other causes of fat accumulation should be excluded such as the excessive consumption of alcohol (women ≤20 g/d, men ≤30 g/d). Moreover, other causes of secondary macrovesicular steatosis should be explored such as parenteral nutrition, hepatitis C, Wilson's disease, states of hunger, lipodystrophy, abetalipoproteinemia, and drugs (e.g., methotrexate, amiodarone, and steroids). Furthermore, clinicians should search for other origins of microvesicular steatosis such as Reye's syndrome, acute fatty liver of pregnancy, HELLP syndrome, metabolic disorders (e.g., lecithin-cholesterol-acyltransferase deficiency), and the use of drugs (e.g., antiretroviral drugs or valproate) [1].

Notably, NAFLD is considered to be the hepatic manifestation of metabolic syndrome, as it is strongly associated with insulin resistance (IR), central obesity, reduced glucose tolerance, type 2 diabetes mellitus (T2DM), arterial hypertension, and hypertriglyceridaemia [2].

However, when we refer to NAFLD, a wide spectrum of disorders is included, ranging from simple nonalcoholic fatty liver (NAFL) to nonalcoholic steatohepatitis (NASH); these disorders are characterised not only by an inflammatory condition, ballooning degeneration, necroapoptosis, and fibrosis but also by a relevant impact on morbidity and mortality [1].

In this review, we analyse the relationship between the complex molecular aspects of NASH and the most interesting and promising treatment options. The literature search included published articles (peer-reviewed original, review, and meta-analyses) with a strong priority for randomised controlled trials (RCT). The search terms included “NAFLD and therapy,” “NASH and therapy,” “NAFLD and treatment,” “NASH and treatment,” “NAFLD and drug,” and “NASH and drug.”

### 1.2. Epidemiology

In the last several years, NAFLD has continued to increase worldwide and is the most common reason for irregular liver tests in both developed and emerging countries [3]. Today, in both Europe and the United Kingdom, NAFLD represents the most frequent cause of chronic liver disease [4, 5]. It was reported that NASH affects approximately 1% of the European and North-American population, and longitudinal studies demonstrated that the histological patterns worsen in approximately one-third of patients who are at risk of progression to cirrhosis [6]. In the United States (US), according to the “National Health and Nutrition Examination Survey,” the proportion of NAFLD among chronic liver diseases increased from 47% to 75% between 1988 and 2008. The rationale for this increase is most likely an increase in metabolic risk factors. In fact, in the same time period, the prevalence of obesity, visceral obesity, T2DM, IR, and arterial hypertension increased [7], and it is well known that patients with NAFL are also more likely to be obese (from 30% to 100%) and have T2DM (from 10% to 75%) [7].

According to the “Organization for Economic Cooperation and Development,” in 2000, 11.5% of German adults were obese, while ten years later, the percentage increased to 14.7% [8]. It was also reported that, in Europe, approximately one-third of adults exhibit a pattern of NAFLD [5]. The prevalence ranges between 13.9% and 26.6% and is based on whether the newest technology (i.e., ultrasound) is used in the screening [9]. However, if we analyse several subgroups such as patients who are affected by T2DM, we find that 44% of patients exhibit a pattern reminiscent of NAFLD [5]. Interestingly, for reasons that are not completely clear, an increase in the prevalence of NAFLD was recently cited among younger (e.g., 12.8% in Australia) and older people (35% in Netherlands) [10, 11].

When the attended variance that is related to the diagnostic methods is considered in the available studies, the global prevalence of NAFLD ranges from 6% to 33%, while the prevalence of NASH is notably lower (3%–5%) [12].

Although many epidemiological studies have shown that NAFLD is strictly connected to an unhealthy diet and sedentary behaviours [13], metabolic liver disease can also be found in nonobese populations [14]. Specifically, Caldwell et al. [15] reported that both ethnicity and genetic polymorphisms could play a central role in the development of this disease.

### 1.3. Molecular Aspects

Traditionally, the pathogenesis of NAFLD was conceived as a “two-hit” process [16]: (a) the accumulation of lipids in hepatocytes promotes IR, which is the key factor for the development of hepatic steatosis; (b) the consequences are an increase in proinflammatory cytokines and adipokines, mitochondrial dysfunction, oxidative stress, and endoplasmic reticulum stress, which lead to hepatocyte injury, inflammation, and fibrosis. In the past few years, many authors [17, 18] have reported that NASH is associated with a significantly worse outcome with respect to simple steatosis. Indeed, it has been suggested that NASH and simple steatosis should be considered two separate diseases. After consideration of the progressive form of NAFLD, which is definitively represented by NASH, a new and more realistic model was proposed: the “multiparallel hits” hypothesis [19]. The main concept that has emerged from this new idea is that different events occur that in turn determine liver damage, but these events take place in parallel, not consecutively. Remarkably, all of the events that favour the pathogenesis of NASH are potentially therapeutic targets. We can consider the following elements as the main pathological protagonists: IR, oxidative stress, gut, adipose and pancreas tissues, altered lipid metabolism, bile acids, gut microbiota, and bacterial endotoxins. It is important to underline that all the mechanisms of damage that are involved in the pathogenesis of NASH enhance two conditions that are central in the progression of NASH: IR and systemic chronic inflammation. The molecular basis of IR is the result of both genetic and nongenetic mechanisms. However, IR initiates a vicious circle that leads to inflammation, hypercoagulability, and atherogenesis. Interestingly, IR develops in the vasculature earlier than in muscle, liver, or adipose tissue, and this explains the high cardiovascular risk that can be found in patients with IR [20]. Tumour necrosis factor-alpha (TNF-*α*) plays an important role in the development of an inflammatory state, determines apoptotic and fibrogenic reactions, and regulates IR [21]. In addition, oxidative stress is considered to be an important stage in the development of NASH. Oxidative stress is due to mitochondrial dysfunction and represents a true turning point for many investigators. The loss of electrons from complexes I and III in the mitochondrial electron transport chain can combine with oxygen, which generates reactive oxygen species; these promote damage to the DNA, lipid membranes, and proteins [22, 23]. Inflammatory mediators that are derived from various tissues such as gut and adipose tissue seem to play a role in the cascade of inflammation and fibrosis of NASH. Within the adipose and liver tissues, increased lipid storage, lipogenesis, and (adipo)cytokine synthesis occur as stress signals for the endoplasmic reticulum (ER). Interestingly, altered adipokine profiles have been suggested to play a pivotal role in the progression of NASH [24]. Glucose-dependent insulinotropic peptide (GIP) and glucagon-like peptide-1 (GLP-1) are incretin hormones that are released by the gastrointestinal tract in response to nutrients. The impairment of incretins, which is observed classically in T2DM, seems to have a role in the development of NASH because it enhances IR and fat accumulation [25]. Bile acids are other significant regulators of glucose homeostasis through many signalling pathways that regulate the metabolism of both glucose and cholesterol. Notably, in conditions such as T2DM, the composition of bile acids is altered and a decrease in the secretion of bile in the gut is observed; consequently, a reduction in the secretion of GLP-1 and impairment of glucose homeostasis may occur. Remarkably, patients with NAFLD can show a hyperinsulinemic state that definitively promotes lipogenesis and hepatic lipid deposition and may also accelerate the development of liver disease [26, 27]. Many authors have suggested a significant connection between altered cholesterol homeostasis and hepatic free cholesterol accumulation as a prompt for the pathogenesis of NASH [28, 29]. Altered lipid metabolism is one of the main stages of the pathogenesis of NASH, which leads to the accumulation of intermediate products such as diacylglycerol and phospholipids (e.g., sphingolipids and ceramides); these are directly associated with fatty acid-induced toxicity and IR. These metabolites promote the activation of many kinases that negatively regulate the insulin pathway [30]. Indeed, the accumulation of free cholesterol in the ER membrane decreases its fluidity and determines cellular stress and apoptosis [31, 32]. The role of gut microbiota in the pathogenesis of NASH has generated a growing interest as modifications in its composition could increase the permeability of the gut and the translocation of bacterial endotoxins, which may promote systemic inflammation and IR [33]. Bacterial endotoxins, such as lipopolysaccharide (LPS), are a significant cause of hepatic neutrophil infiltration in the NASH subjects [34]. Even if the LPS seems to have a relevant role in the pathogenesis of NASH, the underlying mechanisms are uncertain. Imajo et al. [35], through a mice-model study, demonstrated that the upregulation of CD14 in Kupffer cells by leptin-mediated signaling can determine hyperreactivity against the endotoxins. Indeed, the authors showed that hyperresponsivity against low-dose LPS, typically found in the high-fat diet mice, can favour the NASH progression, in terms of both inflammation and fibrosis.

## 2. Standard of Care: Lifestyle Interventions

NAFLD and NASH are linked to excess body weight, an unhealthy diet, and inactive behaviours [13, 36, 37]. Notably, disease progression from NAFL to NASH is basically stated by obesity, T2DM, and metabolic syndrome, all of which are associated with unhealthy activities [13]. According to this view, all guidelines concur that lifestyle modifications are the first-line approach to manage patients with NAFLD and NASH [38–41]. However, while diet and exercise guidelines for conditions such as T2DM and cardiovascular disease are well established, no guidelines exist that indicate the ideal diet and exercise modalities. Nevertheless, it is well known that lifestyle coaching should involve a personalised diet, physical activity, and cognitive-behaviour therapy [42]. In fact, diet and physical activity could support weight loss, the recovery of liver enzymes, and an improvement in histological alterations [43].

According to Promrat et al. [44], who reported interesting data from a RCT, the main targets in patients who are affected by NASH come from the “US Diabetes Prevention Program” and are a weight loss of 7% and 150 minutes/week of physical activity [45]. The authors enrolled patients with NASH and evaluated the effects of lifestyle intervention using a combination of diet, exercise, and behaviour therapy. After 48 weeks of treatment, patients who received a lifestyle intervention lost an average of 9.3% of their weight versus 0.2% in the controls and demonstrated a significant reduction in “NAFLD activity score.” Moreover, the score improvement correlated significantly with weight reduction. In fact, a weight loss of 7% significantly decreased fat accumulation and improved necroinflammation even if a significant effect on fibrosis was not shown [44]. In a meta-analysis in 2010, Musso et al. [46] confirmed that significant weight loss is safe and leads to better histological and metabolic parameters in patients with NASH. In particular, a 5% weight loss seems to decrease liver steatosis and to ameliorate metabolic parameters, but higher weight loss is likely necessary to downgrade the necroinflammation and the overall disease activity. Notably, a gradual weight loss (<1.6 kg/week) should be recommended because faster weight loss might worsen the liver injury [47]. Eckard et al. [48] conducted a RCT that involved 56 patients with a biopsy-proven diagnosis of NAFLD. Subjects were assigned to 1 of 4 lifestyle modification subgroups for 6 months: standard care, low-fat diet and moderate exercise, moderate-fat/low-processed-carbohydrate diet and moderate exercise, or moderate exercise only. All subgroups demonstrated a decrease in the “NAFLD activity score” over the 6-month period with no significant difference between the subgroups. In addition, in all groups a significant decrease was observed in the Brunt grade and in the levels of ALT and AST. Among patients with NASH at baseline, 53% improved their Brunt grade or stage classification, and 25% had no criteria for NASH at 6 months. Interestingly, no subgroup showed a relevant weight loss greater than 5%. In this regard, physical activity, if characterised by aerobic and resistance training, shows an independent positive effect in the decrease of fat in the liver, regardless of the weight loss [49, 50]. Moreover, it should be considered that patients with NAFLD/NASH are often at a high cardiovascular risk, and physical activity, which is expected to improve cardiorespiratory fitness, is important to decrease this hazard [51, 52]. In a thorough review and analysis, Peng et al. [53] reported that the available data are not enough to draw any conclusive results on the proper lifestyle programme and that RCTs are necessary for the evaluation of the beneficial and harmful effects of weight reduction.

Centis et al. [42] analysed the motivation of patients with metabolic liver disease when they changed their habits. The authors demonstrated that a large number of subjects experienced an inadequate inclination to change, particularly with regard to physical activity. This low level of readiness highlights the importance of the use of individually tailored techniques. Notably, in this context, behavioural counselling represents a central part of the therapeutic approach as it affords patients with the information, self-efficacy, critical thinking skills, and tools to realise a better lifestyle so that their prognosis improves.

The lifestyle approach has at least two limits: the compliance of the patients and the difficulty to present a unique and clear scientific vision. In fact, lifestyle modifications can be difficult to put into practice by the patient due to lack of compliance or physical disability. Still, the best solution in terms of nutrients for weight decrease and maintenance remains unclear. Many accessible studies do not report nutrient intake or physical activity and only record little patient information. Studies of the lifestyle interventions in patients with NAFLD/NASH show significant heterogeneity in the enrolled subjects, and, thus, they report limited details on the adherence to the lifestyle changes [54]. For all of these reasons, more scientific energy should be devoted to the improvement of this strong therapeutic tool (see Table 1).

## 3. First-Line Drug: Vitamin E

Vitamin E (RRR-*α*-tocopherol) is an important lipid-soluble antioxidant that is able to scavenge free radicals and avoid lipid peroxidation. In recent years, the therapeutic effects of vitamin E supplementation on NAFLD and NASH have been investigated [55–57], and today, vitamin E should be considered as a first-line pharmacotherapy for patients with biopsy-proven NASH who do not have diabetes. On the contrary, vitamin E is not recommended for the treatment of NASH in diabetic patients, or in cases of NAFLD without liver biopsy, NASH with cirrhosis, or cryptogenic cirrhosis [41].

In a recent meta-analysis, Ji et al. [58] reported that vitamin E supplementation might improve transaminase levels in patients with NASH, which confirms the therapeutic potential of vitamin E [58]. Sanyal et al. [56] developed what is certainly the most relevant RCT with regard to the use of vitamin E in patients with NASH. In their trial of pioglitazone versus vitamin E versus placebo in which 247 adults with NASH were enrolled, vitamin E supplementation (800 IU daily) for 96 weeks significantly improved steatosis and inflammation and resolved the ballooning seen in adult patients with NASH who have aggressive disease. Specifically, patients in the vitamin E arm showed a significantly better histological improvement compared with those who received the placebo. The transaminase level was decreased in patients if it is assumed that both vitamin E and pioglitazone were connected to the reductions of hepatic steatosis and lobular inflammation. Notably, neither vitamin E nor pioglitazone was associated with a significant improvement in fibrosis, and pioglitazone led to an increase in weight compared with vitamin E or placebo.

Validation is needed for the use of vitamin E in children, and long-term RCTs are required to assess the long-term efficacy and safety of vitamin E [59]. Finally, it should be mentioned that a published meta-analysis [60] has suggested that a high dose of vitamin E (>400 IU daily) may increase the risk for all-cause mortality. Nevertheless, in this meta-analysis, which included 19 trials, high-dosage trials (≥400 IU/d) were smaller, on average, compared with others, and the studied population was not homogeneous (see Table 2).

## 4. Second-Line Option: Pioglitazone

As IR is a well-known hallmark in the pathogenesis of NASH, thiazolidinediones (pioglitazone and rosiglitazone) were reported as possible therapeutic options.

Sanyal et al. [56] previously reported that pioglitazone can lead to a decrease in AST and ALT levels and, from a histological point of view, can decrease steatosis and lobular inflammation. Other authors had previously described the effects of pioglitazone on NASH with partial and doubtful results. In two small, noncontrolled studies, Promrat et al. and Lutchman et al. [61, 62] suggested that 48 weeks of pioglitazone (30 mg/day) may improve NASH both biochemically and histologically. However, they also reported that weight gain is a major side effect of the long-term use of the pioglitazone and that this may significantly limit its use. Through two stronger, double-blind RCTs, Belfort et al. [63] and Aithal et al. [64] demonstrated that pioglitazone (45 mg daily for 24 weeks in the first study and 30 mg daily for 48 weeks in the second) not only caused weight gain but also enhanced tissue inflammation and fibrosis. In 2011, Musso et al. [65] reported that pioglitazone could positively improve liver histology and the cardiometabolic profile in the context of NASH, but it also demonstrated the same impact with respect to weight loss.

With regard to rosiglitazone, in 2008, Ratziu et al. [66] published a RCT that suggested that the positive effect of this drug on the AST/ALT levels and on liver steatosis led to weight gain but had no significant effect on fibrosis.

In 2012, a meta-analysis on the use of pioglitazone and rosiglitazone in the treatment of NASH was performed [67]. The authors reported that thiazolidinediones might decrease ALT levels and improve histological parameters and that pioglitazone might reverse fibrosis in NASH; however, this latter effect represents the most important point of interest.

According to a recently published review article that was conducted by an expert panel from the “Chilean Gastroenterological Society” and the “Chilean Hepatology Association” [68], pioglitazone together with vitamin E is a proven pharmacological choice for patients with biopsy-proven NASH, although evidence on its long-term safety and efficacy is lacking. Additionally, the guidelines of the American Association for the Study of Liver Diseases [41] indicate pioglitazone as a possible therapeutic drug for the treatment of NASH. However, the vast majority of patients enrolled in the available studies did not have a diagnosis of T2DM, and, therefore, data about its long-term efficacy and safety are not available (see Table 3).

## 5. Broken Promises

### 5.1. Metformin

It is well known that metformin can positively affect IR, which is a cornerstone of the pathogenesis of NASH. Consequently, the possible role of this drug in the treatment of metabolic liver disease has been extensively studied. In 2004, Nair et al. [69] published a small open-label trial and reported that a three-month therapy with metformin was able to decrease the levels of ALT/AST but that this positive effect was only transitory. Remarkably, the authors reported a lack of significant improvement in both liver inflammation and fibrosis. In a small pilot study, Loomba et al. [70] showed that 48 weeks of metformin (2.000 mg/day) did not have any significant effect on the histological features of NASH. In the following year, Haukeland et al. [71] developed a more significant study. The authors enrolled 48 patients with biopsy-proven NAFLD in a RCT with metformin or placebo for 6 months. The absence of histological changes as a result of the drug was confirmed, but a decline in the serum levels of both lipids and glucose was observed. Furthermore, in a RCT with metformin (500–1000 mg daily for 12 months) versus placebo, Shields et al. [72] corroborated the absence of a significant positive effect on liver histology and also the lack of a significant weight loss. A systematic review of 8 RCTs showed no favourable effects of metformin on the histology of NASH although it can lead to weight loss [73]. Indeed, although metformin can be used in diabetic patients with NASH, no evidence exists to support its efficacy in terms of the histology of NASH [74] (see Table 4).

### 5.2. Ursodeoxycholic Acid

Ursodeoxycholic acid (UDCA) has been widely studied for the treatment of NASH for at least three reasons: its antiapoptotic properties and its capacity to decrease TNF-*α* and ER stress [75, 76]. Lindor et al. [77] performed a RCT of 166 patients with liver biopsy-proven NASH who were treated with 13–15 mg/kg daily of UDCA or placebo for 24 months. The authors found no difference between the two arms in terms of histological improvement. Still, because good outcomes that resulted from the use of higher doses of UDCA were reported in liver disease [78], some authors tried to use a high dosage approach. Leuschner et al. [79] enrolled 185 patients with a histological diagnosis of NASH in a RCT and offered them high-dose UDCA (23–28 mg/kg daily) or placebo for 18 months. The authors reported a positive effect of the drug only on lobular inflammation but did not demonstrate any positive effects on fibrosis or on laboratory data. Ratziu et al. [80] developed a RCT that included 126 subjects with biopsy-proven NASH who were treated with 28–35 mg/kg daily or placebo for 12 months. The authors demonstrated that a high dose of UDCA was more effective in terms of a decrease in ALT and indirect indexes of fibrosis and IR, but the results were hampered by a lack of serial biopsies. This latter aspect makes the results of this study insufficient to justify the use of UDCA in patients with NASH. This assumption is clearly reported in the editorial by Haedrich and Dufour [81], who assumed that UDCA given in a monotherapy at the usual dose shows no positive effects in patients with NASH, and exerts only minor effects at a higher dose. Moreover, the potentially damaging adverse events that may occur during high-dose UDCA treatment should be noted and considered (see Table 5).

### 5.3. Statins

Hyperlipidaemia (hypertriglyceridaemia, hypercholesterolaemia, alone or together) is often associated with obesity and T2DM and is present in 20–80% of patients who are diagnosed with NASH [82, 83]. In NASH, the plasma levels of lipids are increased and the accumulation of triacylglycerol in the liver is a hallmark of the pathogenesis of this disease [84]. In the same vein, it was reported that statins, which are inhibitors of hydroxymethylglutaryl-coenzyme A reductase, might be a valid therapeutic option to decrease intrahepatic cholesterol and to improve the abnormal metabolism of lipids [85].

In a well-written review that was published last year, Eslami et al. [86] reported that only two studies concerned the use of statins in patients with NAFLD/NASH. These studies showed the prerequisites for a concrete evaluation although none of the available ones analysed the following relevant outcomes: liver-related morbidity, liver-related mortality, or all-cause mortality.

The first study was a trial that compared atorvastatin versus fenofibrate versus a combination of the two interventions [87]. In this study, no differences with regard to plasma liver enzyme activities or imaging findings were found, but when these two outcomes were evaluated together, atorvastatin appeared to be better than fenofibrate.

The other study was a small pilot trial that evaluated simvastatin versus placebo [88]. The authors reported no significant effects of simvastatin versus placebo in terms of the levels of AST and ALT and in terms of liver histology.

The known side effects of statins, such as myositis, myalgia, rhabdomyolysis, elevations in aminotransferases, and acute renal failure, are well described. However, the number of adverse events was not significantly different between groups in the two cited studies. Again, after a consideration of other works that have proposed the use of statins in the context of NASH, no adverse events were reported [89–91].

Indeed, statins, which seem to be safe in patients with NASH, can be considered in particular situations such as in patients with NAFLD/NASH who have increased serum cholesterol levels. In this case, the increased cardiovascular risk can independently indicate the use of these drugs [92] (see Table 6).

### 5.4. Pentoxifylline

Pentoxifylline is a methylxanthine derivative and nonspecific phosphodiesterase inhibitor that is usually used in the treatment of intermittent claudication for its effects in the improvement of red blood cell flexibility, decrease in blood viscosity, and enhancement of aerobic glycolysis and oxygen consumption in ischaemic tissues [93]. Moreover, it was demonstrated that pentoxifylline decreases gene transcription of TNF-*α*, which influences multiple steps of the cytokine/chemokine pathway [94]. Lee et al. [95] proposed a small RCT that did not involve a histological analysis; they compared pentoxifylline (1200 mg daily for 3 months) and placebo and showed that the only positive effect of the drug was on the AST level. In 2009, Rinella et al. [96] published a RCT in which patients with NASH were treated with pentoxifylline (1200 mg daily for 12 months) or placebo; the drug exerted a significant effect only on steatosis and cellular ballooning. A few years later, Zein et al. [97] developed a larger RCT that also included the histological analysis. Pentoxifylline (1200 mg daily for 12 months) improved steatosis and tissue inflammation compared with the placebo but did not show a significant influence on fibrosis. In the same year, van Wagner et al. [98] published a RCT with a similar structure as the previous trial and reported no significant positive action of the drug either on the biochemical data or on the liver histology. In 2011, a systematic review that concerned the use of pentoxifylline in patients with NASH [99] demonstrated that pentoxifylline seems to decrease both AST and ALT levels but cannot influence cytokine levels and histological aspects of NASH (see Table 7).

### 5.5. Orlistat

Orlistat is an enteric lipase inhibitor that was evaluated as a possible therapeutic choice for patients with NAFLD/NASH since it was reported that it might lead to weight reduction, decreased free fatty acid flux to the liver, and improved insulin sensitivity, without hepatotoxic adverse effects. This drug was studied in two RCTs in combination with lifestyle modification. Zelber-Sagi et al. [100] proposed a RCT in which patients were randomised to receive either orlistat (120 mg, 3 times daily for 6 months) or placebo; with this drug, patients showed an improvement in ALT levels, weight, and hepatic steatosis. However, these authors did not analyse the modifications in liver histology. In another RCT, Harrison et al. [101] randomised patients with biopsy-proven NASH to a 1400 kcal/day diet and 800 IU vitamin E/day with or without orlistat (the same dose with respect to the previous study but for 9 months) and demonstrated no significant improvement in AST/ALT levels, weight, insulin sensitivity, and liver histology. Notably, patients who lost 5% or more of their weight also experienced a reduction in steatosis, IR, and plasma glucose, but only those subjects who lost 9% or more of their weight experienced an improvement in necroinflammation as well (see Table 8).

## 6. Emerging Treatment Options 

### 6.1. Vitamin E Associations

After consideration of the articulated and multifactorial pathogenesis of NASH, it is reasonable to think that the combination of multiple drugs that are directed at different targets may lead to a gain in terms of effectiveness. The association of one molecule with one other drug with a certain degree of scientific evidence is reasonable. Indeed, vitamin E supplementation in combination with other drugs has been widely used. In 2006, Dufour et al. [102] published a RCT in which patients with NASH were randomly allocated to receive UDCA (12–15 mg/kg daily) with vitamin E (800 IU daily), UDCA with placebo, or placebo alone. The combination of UDCA and vitamin E led to better serum levels of alanine aminotransferase (ALT)/aspartate aminotransferase (AST) and liver steatosis in comparison with UDCA monotherapy. Notably, the authors did not exclude the possibility that, at a higher dosage, UDCA might be more effective, and the absence of an arm that included vitamin E plus placebo did not provide information about the efficacy of vitamin E monotherapy. The same study group [103] analysed the effects of UDCA-vitamin E on adipokines and apoptosis of hepatocytes, which demonstrated that this combination improved the levels of transaminases as well as liver histology and decreased cellular apoptosis. Pietu et al. [104], through a large retrospective study, reported that the well-tolerated combination of UDCA and vitamin E significantly improved ALT, AST, and gamma-glutamyl transpeptidase levels, although the authors did not evaluate the histological impact of this therapeutic approach. In this context, a RCT that compares vitamin E monotherapy with vitamin E plus UDCA might be useful.

It was reported that the association of vitamins E and C might reduce fibrosis in patients with NASH [105] as the combination of vitamin E with other antioxidants would lead to a regeneration of the oxidised form of the vitamin [106]. However, the lack of a RCT precludes the ability to accurately evaluate this therapeutic choice [107] (see Table 9).

### 6.2. Angiotensin II Receptor Blockers

The role of the renin-angiotensin system (RAS) in renal and cardiovascular responses is well known. Angiotensin II receptor blockers (ARBs) are a consolidated family of antihypertensive drugs. However, other tissues produce RAS components that are regulated independently from RAS in the circulatory system. In the liver, chronic injury upregulates RAS in local tissues, which seems to contribute to the vicious cycle of steatosis-necroinflammation-fibrosis. Indeed, many authors suggest that ARBs may be useful in the treatment of NAFLD/NASH. It was reported that losartan has an antifibrotic effect and improves steatosis and necroinflammation in patients affected by NASH [108, 109]. Interestingly, Torres et al. [110] proposed the use of losartan for NASH in a RCT (rosiglitazone versus rosiglitazone and metformin versus rosiglitazone and losartan) but found no significant results. On the contrary, the use of telmisartan shows the longest terminal elimination half time and the greatest affinity for the angiotensin II receptor type 1 among the ARBs. It was reported in animal models that this drug reduced weight, improved hyperinsulinaemia, and decreased triglycerides, steatosis, fibrosis, and liver macrophage infiltration [111–114]. Interestingly, Clemenz et al. [115] also indicated telmisartan as a partial PPAR-alpha agonist, which suggests that it causes a reduction of circulating and hepatic triglycerides. Georgescu et al. [116] first demonstrated that telmisartan (20 mg daily) was better than valsartan for the improvement of IR and liver histology (i.e., steatosis, lobular inflammation, ballooning, and fibrosis), and its action on IR was confirmed by Miura et al. [117]. With regard to its safety, Schumacher and Mancia [118] retrospectively analysed 50 studies on telmisartan and showed that it has a placebo-like tolerability. Indeed, among the ARBs, telmisartan is the most promising for the treatment of NASH in terms of both safety and efficacy on inflammation and fibrosis; however, the lack of a large RCT precludes the use of this drug as a consolidated option (see Table 10).

### 6.3. Pre- and Probiotics

It was demonstrated that bacterial overgrowth in the bowel is present in 50% of patients with NASH [119]. Moreover, a high-fat diet-induced obesity is related to changes in the composition of intestinal bacteria [120]. Indeed, modifications of the intestinal bacterial content might be involved in the pathogenesis of NASH through the enhancement of intestinal permeability, the direct activation of inflammatory cytokines, and the improvement of the absorption of endotoxins [121].

Aller et al. [122] proposed a double-blind RCT in which patients with metabolic liver disease were treated with 500 million* Lactobacillus bulgaricus* and* Streptococcus thermophilus* or placebo. Patients in the drug arm showed an improvement in ALT/AST levels but did not show effects in terms of anthropometric parameters and cardiovascular risk factors. Malaguarnera et al. [123] evaluated the effects of* Bifidobacterium longum*, fructooligosaccharides, and lifestyle modification versus lifestyle modification alone, for the treatment of NASH. In this RCT, the authors showed that* Bifidobacterium longum* together with fructooligosaccharides significantly decreased TNF-*α*, C-reactive protein, AST levels, IR, serum endotoxin levels, steatosis, and the “NASH activity index.” Finally, Wong et al. [124] tested a Lepicol probiotic formula (*Lactobacillus plantarum*,* Lactobacillus delbrueckii*,* Lactobacillus acidophilus*,* Lactobacillus rhamnosus*, and* Bifidobacterium bifidum*) in a small trial to cure patients with biopsy-proven NASH. The authors obtained a reduction in liver fat, as measured by proton-magnetic resonance spectroscopy, and a decrease in AST levels.

In a meta-analysis published in 2013 [125], it was suggested that probiotics might significantly decrease the levels of ALT, AST, total cholesterol, TNF-*α*, and IR and exert virtually positive effects on the pathogenesis of NASH (see Table 11).

### 6.4. Fatty Acid-Bile

Bile acids, but not UDCA, control metabolism by binding to the nuclear hormone receptor farnesoid X and to a transmembrane bile acid receptor. Stimulation of the farnesoid X receptor could increase insulin sensitivity and decrease both glucose and lipids. Transmembrane bile acid receptors are regulators of glucose homeostasis and lipid metabolism, and activation of these receptors stimulates energy expenditure and protects against obesity. Farnesoid X receptor agonists were proposed as possible treatment options for metabolic disorders such as T2DM, hypertriglyceridaemia, certain cases of cholestasis, and cholesterol gallstone disease [126]. Indeed, bile acids are metabolic integrators and are not solely regulators of bile-acid homeostasis. As a consequence, non-UDCA bile acids should also be considered in the context of NAFLD and NASH [127]. Similarly, Mudaliar et al. [128] developed a double-blind, placebo-controlled, proof-of-concept study on the use of obeticholic acid in patients with NASH and T2DM. Six weeks of therapy with this drug led to an improvement in IR and a decrease in the indirect index of liver inflammation and fibrosis. More recently, Neuschwander-Tetri et al. [129] developed a multicentre, double-blind, placebo-controlled trial, enrolling patients affected by NASH. Authors proposed a 72-week treatment with obeticholic acid (25 mg daily, orally). Patients in the drug arm with respect to those in the placebo one showed an improvement in the main histological features of NASH. However, as the same authors reported, data about the long-term benefit and safety of this drug are today not available (see Table 12).

### 6.5. Omega-3

It was reported that natural fatty acids such as long-chain omega-3 (LCn-3) polyunsaturated fatty acids (PUFAs), including eicosapentaenoic acid and docosahexaenoic acid, show bioactive properties; therefore, these are a potential treatment option for patients with NASH [130]. LCn-3 PUFA supplementation is a promising treatment for patients with NAFLD/NASH as it can affect different aspects of this complex disease. Animal studies and preliminary clinical trials have demonstrated that purified n-3 supplementation or fish intake might prevent or reverse the rate of NASH [131]. Notably, the therapeutic effects of LCn-3 PUFAs were verified in three studies, two of which included patients with NAFLD [132, 133] and one of which evaluated subjects with NASH [134]. In the latter study, 1 year of treatment with eicosapentaenoic acid significantly improved steatosis (imaging and histological) as well as the levels of AST/ALT, cholesterol, iron, and free fatty acids but did not have any effect on IR. Markedly, the lack of a control group and the small sample size do not allow for significant conclusions (see Table 13).

### 6.6. L-Carnitine

L-Carnitine is a modulator of mitochondrial free fatty acid transport and oxidation that has demonstrated a significant effect on oxidative stress, activation of immune cells, and the integrity of the epithelial barrier. Interestingly, a potential therapeutic value was reported for the use of L-carnitine supplementation in cases of intestinal inflammation [135]. On the assumption that liver steatosis is a common pattern of both hepatitis C and NAFLD/NASH, a total of 70 affected patients were randomly assigned to receive interferon and ribavirin alone or plus carnitine; the patients in this second arm showed a relevant decrease in steatosis [136].

In a high quality RCT conducted by Malaguarnera et al. [137], patients with NASH and control subjects were randomly treated with L-carnitine plus modified diet or modified diet alone. L-Carnitine plus lifestyle intervention for 6 months improved steatosis, the “NASH activity score,” AST/ALT levels, IR, plasma glucose, and total and low-density lipoprotein cholesterol.

The results of this supplementation therapy are very interesting and need to be confirmed in a large and well-structured RCT that tests the efficacy of L-carnitine used alone or with other drugs (see Table 14).

## 7. Conclusions and Future Perspectives

NASH will continue to be the most common liver-related health problem in the future. In fact, the emerging direct antiviral therapies for hepatitis C and the well-consolidated drugs for hepatitis B will allow these liver diseases to be treatable in a vast majority of cases. On the contrary, the best treatment modalities for fatty liver disease together with alcohol-related disease have yet to be elucidated. Today, the incidence of NAFLD and NASH is increasing in industrialised and emerging countries, and this increase is strictly connected to the increase in obesity, visceral obesity, T2DM, IR, and arterial hypertension. In the pathogenesis of NASH, according to the most recent view, many events occur in parallel, and all are potential therapeutic targets. The principally studied actors are the following: IR, oxidative stress, gut, adipose and pancreas tissues, altered lipid metabolism, bile acids, gut microbiota, systemic chronic inflammation, and genetic patterns.

Today, the standard of care for the treatment of NASH is lifestyle modification. However, its effect on liver histology in patients with NASH warrants further evaluation, as the lack of evidence regarding the optimal dietary nutrient composition and exercise is a significant ongoing problem. Moreover, a vast majority of patients show a lack of compliance with diet and exercise programs due to their attitudes or physical inability. Currently, the first-line pharmacological approaches for NASH are vitamin E and pioglitazone as they are unique drugs that have provided a sufficient degree of evidence in terms of efficacy. In particular, both vitamin E and pioglitazone improve the levels of transaminases, fat accumulation, and liver inflammation. However, these two drugs also display evident limits. In particular, vitamin E has not been proven to have an effect on fibrosis or on the long-term morbidity and mortality, and its possible use is limited to patients without T2DM. With regard to pioglitazone, its main restriction is its negative impact on weight. In addition, the safety of this drug remains uncertain as data about the long-term use of vitamin E or pioglitazone are not available. On the one hand, many drugs have been tested through RCTs, which showed only a few relevant results in terms of efficacy, such as metformin, UDCA, statins, pentoxifylline, and orlistat. On the other hand, many emerging treatment options can be found in the literature. Among them, in our opinion, the most attention should be focused on telmisartan, a safe antihypertensive drug. This drug, although it has not yet been tested in a large and well-structured RCT, seems to have a significant impact on IR, liver steatosis, inflammation, and liver fibrosis according to preliminary studies. Additionally L-carnitine is a particularly promising supplement that could be evaluated alone or in association with other drugs.

In the complex establishment of a study on NASH, some aspects should be improved with respect to the available papers. Lifestyle intervention should be clearly defined as it needs to represent a clear point of comparison with respect to newly proposed drugs. In addition, we suggest that the lifestyle intervention itself has a significant potential to expand as specific and widely accepted alimentary, exercise, and counselling indications do not actually exist. Future RCTs need to include histological endpoints and an adequate duration and should also consider patients with advanced fibrosis. Notably, an adequate follow-up period will allow for the assessment of the long-term efficacy and safety of the proposed treatments and for the evaluation of strong outcomes such as the development of cirrhosis and hepatocellular carcinoma, the morbidity and liver, cardiovascular, and all-cause mortality.

Today, many authors continue to develop RCTs with different agents in order to cure patients with NASH and NASH-related fibrosis [138]. However, because multiple parallel roads characterise the complex pathogenesis of NASH, we suggest the evaluation of the combination of different drugs that could act synergistically on the onset and progression of this disease. According to this view, the development of individualised treatment would be opportune. Consequently, the experimental use of vitamin E and UDCA without obvious positive results was a good course of action.

In conclusion, many aspects with regard to therapy for patients with NASH should be implemented. In particular, lifestyle interventions should be clearly defined and optimised, the individualisation of a pharmacological approach with associations of different drugs might be tested, and large RCTs with histological outcomes and long-term observations should be developed.

## Figures and Tables

**Table 1:** 

Effects on	Weight loss	IR	AST/ALT	Histology		Long-term morbidity	Long-term mortality
				Inflammation	Fibrosis		
Lifestyle interventions	Yes	Yes	Yes	Yes	Unproven	Unproven	Unproven

**Table 2:** 

Effects on	Weight loss	IR	AST/ALT	Histology		Long-term morbidity	Long-term mortality
				Inflammation	Fibrosis		
Vitamin E	No	No	Yes	Yes	No	Unproven	Unproven

**Table 3:** 

Effects on	Weight loss	IR	AST/ALT	Histology		Long-term morbidity	Long-term mortality
				Inflammation	Fibrosis		
Pioglitazone	Opposite	Yes	Yes	Yes	Yes	Unproven	Unproven

**Table 4:** 

Effects on	Weight loss	IR	AST/ALT	Histology		Long-term morbidity	Long-term mortality
				Inflammation	Fibrosis		
Metformin	Yes	Yes	No	No	No	Unproven	Unproven

**Table 5:** 

Effects on	Weight loss	IR	AST/ALT	Histology		Long-term morbidity	Long-term mortality
				Inflammation	Fibrosis		
UDCA	No	No	No	Yes	No	Unproven	Unproven

**Table 6:** 

Effects on	Weight loss	IR	AST/ALT	Histology		Long-term morbidity	Long-term mortality
				Inflammation	Fibrosis		
Statins	No	No	No	No	No	Unproven	Unproven

**Table 7:** 

Effects on	Weight loss	IR	AST/ALT	Histology		Long-term morbidity	Long-term mortality
				Inflammation	Fibrosis		
Pentoxifylline	No	No	Yes	No	No	Unproven	Unproven

**Table 8:** 

Effects on	Weight loss	IR	AST/ALT	Histology		Long-term morbidity	Long-term mortality
				Inflammation	Fibrosis		
Orlistat	No	No	No	No	No	Unproven	Unproven

**Table 9:** 

Effects on	Weight loss	IR	AST/ALT	Histology		Long-term morbidity	Long-term mortality
				Inflammation	Fibrosis		
Vitamin E/UDCA	No	No	Yes	Unproven	Unproven	Unproven	Unproven

**Table 10:** 

Effects on	Weight loss	IR	AST/ALT	Histology		Long-term morbidity	Long-term mortality
				Inflammation	Fibrosis		
ARBS	Unproven	Yes	Unproven	Unproven	Unproven	Unproven	Unproven

**Table 11:** 

Effects on	Weight loss	IR	AST/ALT	Histology		Long-term morbidity	Long-term mortality
				Inflammation	Fibrosis		
Pre- and probiotics	No	Yes	Yes	Unproven	Unproven	Unproven	Unproven

**Table 12:** 

Effects on	Weight loss	IR	AST/ALT	Histology		Long-term morbidity	Long-term mortality
				Inflammation	Fibrosis		
Fatty acid-bile	Unproven	Unproven	Unproven	Unproven	Unproven	Unproven	Unproven

**Table 13:** 

Effects on	Weight loss	IR	AST/ALT	Histology		Long-term morbidity	Long-term mortality
				Inflammation	Fibrosis		
Omega-3	Unproven	Unproven	Unproven	Unproven	Unproven	Unproven	Unproven

**Table 14:** 

Effects on	Weight loss	IR	AST/ALT	Histology		Long-term morbidity	Long-term mortality
				Inflammation	Fibrosis		
L-Carnitine	Unproven	Unproven	Unproven	Unproven	Unproven	Unproven	Unproven
